# Lung stereotactic body radiation therapy for elderly patients aged ≥ 80 years with pathologically proven early-stage non-small cell lung cancer: a retrospective cohort study

**DOI:** 10.1186/s13014-021-01769-7

**Published:** 2021-02-23

**Authors:** Kenta Watanabe, Kuniaki Katsui, Soichiro Sugiyama, Kotaro Yoshio, Masahiro Kuroda, Takao Hiraki, Katsuyuki Kiura, Yoshinobu Maeda, Shinichi Toyooka, Susumu Kanazawa

**Affiliations:** 1 Department of Radiology, Okayama University Hospital, 2-5-1 Shikata-cho, Kita-ku, Okayama, 700-8558 Japan; 2 Department of Proton Beam Therapy, Okayama University Graduate School of Medicine, Dentistry, and Pharmaceutical Sciences, 2-5-1 Shikata-cho, Kita-ku, Okayama, 700-8558 Japan; 3 Department of Radiological Technology, Graduate School of Health Sciences, Okayama University, 2-5-1 Shikata-cho, Kita-ku, Okayama, 700-8558 Japan; 4 Department of Radiology, Okayama University Graduate School of Medicine, Dentistry, and Pharmaceutical Sciences, 2-5-1 Shikata-cho, Kita-ku, Okayama, 700-8558 Japan; 5 Department of Allergy and Respiratory Medicine, Okayama University Hospital, 2-5-1 Shikata-cho, Kita-ku, Okayama, 700-8558 Japan; 6 Department of Hematology, Oncology, and Respiratory Medicine, Okayama University Graduate School of Medicine, Dentistry, and Pharmaceutical Sciences, 2-5-1 Shikata-cho, Kita-ku, Okayama, 700-8558 Japan; 7 Department of General Thoracic Surgery and Breast and Endocrinological Surgery, Okayama University Graduate School of Medicine, Dentistry, and Pharmaceutical Sciences, 2-5-1 Shikata-cho, Kita-ku, Okayama, 700-8558 Japan

**Keywords:** Clinical pathology, Elderly, Non-small cell lung carcinoma, Radiosurgery, Stereotactic body radiation therapy

## Abstract

**Background:**

Stereotactic body radiation therapy (SBRT) is an established therapy for medically inoperable early-stage non-small cell lung cancer (NSCLC). Many elderly patients are medically inoperable owing to comorbidities. Therefore, SBRT may be a useful therapy for elderly patients. However, the application of SBRT for patients aged ≥ 80 years has not been completely elucidated. Therefore, this study aimed to assess the clinical utility of SBRT for elderly patients aged ≥ 80 years with pathologically proven early-stage NSCLC.

**Methods:**

We retrospectively evaluated the data of patients aged ≥ 80 years with pathologically proven primary NSCLC who underwent SBRT at our institution between January 2009 and March 2020. Treatment outcomes and toxicities were analyzed. We used the Kaplan–Meier method to estimate survival curves and the log-rank test to compare the survival curves. We performed univariate and multivariate Cox regression analyses. *p*-values < 0.05 were regarded significant.

**Results:**

Sixty-four patients (65 lesions) were included, and the median follow-up period was 38.7 (range 3.5–95.7) months. The median age was 82.9 (range 80.0–94.8) years. Sixteen patients were medically operable, and 48 patients were medically inoperable. The prescribed dose of SBRT was either 48 Gy in four fractions or 60 Gy in 10 fractions. The median survival time was 60.0 months (95% confidence interval, 43.5–71.1). The 1-, 3-, and 5-year local control, cancer-specific survival, progression-free survival, and overall survival rates were 98.4%, 98.4%, 81.0%, and 88.9%; 90.1%, 93.7%, 58.9%, and 68.3%; and 87.4%, 83.5%, 38.2%, and 47.5%, respectively. Multivariate analysis revealed that inoperability and solid nodules were the predictors of poor overall survival after SBRT in elderly patients. Two patients (3.1%) had grade 3 radiation pneumonitis, and one patient (1.6%) had grade 5 radiation pneumonitis.

**Conclusions:**

SBRT was feasible in patients aged ≥ 80 years with NSCLC. It achieved good local control with minimal toxicity. SBRT may be beneficial in elderly patients with early-stage NSCLC.

## Background

Primary lung cancer is one of the common life-threatening malignancies and is the main cause of death among all cancers [1]. The proportion of elderly patients with primary lung cancer is predicted to increase owing to the aging of the general population [2, 3]. Therefore, the treatment strategy for elderly patients with primary lung cancer is an important consideration.

Surgery is the standard treatment for early-stage non-small cell lung cancer (NSCLC) [4]. However, no evidence-based standard treatments for elderly patients with NSCLC exist, particularly for those aged ≥ 80 years. Although surgical resection is an effective treatment for elderly patients [5], it is often not feasible in elderly patients aged ≥ 80 years owing to comorbidities.

Stereotactic body radiation therapy (SBRT) is the usual treatment for medically inoperable patients, and it provides good local control rates and induces minimal toxicity [6–11]. Therefore, SBRT may be beneficial in elderly patients. According to a national survey in South Korea, the percentage of patients aged ≥ 80 years receiving SBRT as a treatment modality for early NSCLC increased from 9.4% in 2008 to 28.6% in 2016 [12]. While some studies have revealed that SBRT is an effective and safe treatment modality in elderly patients aged ≥ 80 years [13–15], most studies included patients who were not pathologically confirmed to have NSCLC.

Pulmonary nodules may be benign (e.g., organized pneumonia, tuberculosis, and sclerosing hemangioma) [16, 17]. Radiation therapy to treat these infectious nodules or benign lesions is contraindicated. Therefore, it is desirable to make a definitive diagnosis before cancer treatment. A study reported the outcomes of SBRT for patients aged ≥ 75 years with pathologically proven early-stage NSCLC [18]. This study revealed that SBRT had excellent tumor control rates and low toxicity rates in patients aged ≥ 75 years with proven NSCLC. However, to the best of our knowledge, with respect to the clinical outcome of SBRT, there is no study in which all patients aged ≥ 80 years have been pathologically diagnosed to have NSCLC. Thus, this study aimed to assess the clinical utility of SBRT for elderly patients aged ≥ 80 years with pathologically proven early-stage NSCLC.

## Methods

### Patients

We retrospectively evaluated the data of consecutive patients aged ≥ 80 years at the start of SBRT for T1-2N0M0 (tumor-node-metastasis classification, 8th edition) primary lung cancer at Okayama University Hospital between January 2009 and March 2020. Patients without pathologically proven NSCLC were excluded.

Computed tomography (CT) scan was performed for all patients to stage their lung cancer. On CT, the lesions were classified as ground-glass, part solid, or solid, and tumor size was measured including that of ground glass lesions. Positron emission tomography, if performed, was also used for cancer staging. All tumors were subjected to CT-guided or bronchoscope-guided biopsies to confirm the pathology. Data related to patients’ pretreatment Eastern Cooperative Oncology Group (ECOG) performance status (PS) score and body mass index were collected. The Charlson Comorbidity Index was calculated. Global Initiative for Chronic Obstructive Lung Disease stage was classified according to the results of the pretreatment pulmonary function tests. Board-certified thoracic surgeons or respiratory physicians determined whether the patients could tolerate surgical resection after considering respiratory function, comorbidities, possibility of general anesthesia, and general condition. Patients who could not tolerate surgery were defined as medically inoperable.

### Treatment

Long-scan-time non-breath-hold CT was performed to delineate the internal target volume after immobilization with the Vac-Loc and Hip-Fix systems (CIVCO Medical Solutions, Orange City, IA, USA), and compressing the chest and abdomen. The respiratory motion was reduced by using the aforementioned device and respiratory depression. We used X-ray fluoroscopy to evaluate tumor motion. The planning target volume (PTV) included the internal target volume plus a 5–8-mm margin. A radiation therapy planning system (Xio version 4.8.0; Elekta, Stockholm, Sweden) was used with a superposition dose calculation algorithm with heterogeneity correction. Radiation therapy was delivered using 6-MV photons from a linear accelerator (Primus; Canon Medical Systems, Tochigi, Japan) in multiple coplanar and non-coplanar static ports. We prescribed 48 Gy in 4 fractions for peripheral lesions and 60 Gy in 10 fractions for central lesions or lesions adjacent to the brachial plexus. SBRT was performed daily on weekdays. Until October 31, 2015, the prescribed dose was defined as the dose at the isocenter. Subsequently, the prescribed dose was defined as the dose covering 95% of the PTV (D95). We evaluated toxicities using the Common Terminology Criteria for Adverse Events (version 5.0) [19]. We collected the data on grade ≥ 3 adverse events.

### Statistical analysis

We estimated local control (LC), cancer-specific survival (CSS), progression-free survival (PFS), and overall survival (OS) rates using the Kaplan–Meier method and compared survival curves using the log-rank test. We performed univariate and multivariate Cox regression analyses to determine if either clinical or treatment-related factors could predict LC, CSS, PFS, or OS. Variables with *p*-values < 0.05 in the univariate analysis were retained in the multivariate analysis. We used Stata/IC 16.1 (StataCorp, College Station, TX, USA) to conduct statistical analyses. *p*-values < 0.05 were considered statistically significant.

## Results

### Patient characteristics

In total, 162 consecutive patients (166 lesions) were treated with SBRT at our hospital between January 2009 and March 2020. Among those patients, 64 patients (65 lesions; 45 men and 19 women) aged ≥ 80 years with pathologically proven NSCLC were included in our study. Sixty-three patients had been evaluated using positron emission tomography-CT before the treatment. The median (range) age and ECOG PS were 82.9 (80.0–94.8) years and 1 (0–3), respectively. The median (range) follow-up period after SBRT was 38.7 (3.5–95.7) months. One patient had synchronous primary lung cancer. No patient had interstitial pneumonia before treatment. Patient and tumor characteristics are summarized in Table 1.

**Table 1:** Patient and tumor characteristics

Characteristic	Value
Age (years), median (range)	83 (80.0–94.8)
Sex (n), male/female	45/19
Follow-up (months), median (range)	39 (3.5–95.7)
BMI (kg/m^2^), median (range)	22.8 (16.7–29.4)
BMI distribution (n), < 18.5/18.5–25.0/ > 25 kg/m^2^	5/48/11
ECOG PS (n), 0/1/2/3	28/19/11/6
Operability (n), yes/no	16/48
GOLD stage (n), normal/I/II/III†	24/18/13/4
FEV1.0 (L), median (range) †	1.6 (0.7–2.7)
History of lung operation (n), yes/no	14/50
Charlson Comorbidity Index (n), 2/3/4/5/6/7/9	10/28/12/7/3/2/2
T stage (n), Tis/T1mi/T1a/T1b/T1c/T2a/T2b	2/2/5/26/17/10/3
Maximum tumor diameter (cm), median (range)	2.1 (0.8–4.1)
ITV (mL), median (range)	10.2 (1.1–51.8)
PTV (mL), median (range)	32.4 (9.6–110.6)
Histology (n)	
Adenocarcinoma	43
Squamous cell carcinoma	19
Unclassified NSCLC	3
Tumor opacity (n), pure GGN/part solid GGN/solid	2/18/45
Location (n), central/peripheral	1/64
Location (n), left/right	24/41
Location (n), upper lobe/middle lobe/lower lobe	37/1/27
Prescribed dose (n)	
48 Gy (isocenter)	41
60 Gy (isocenter)	6
48 Gy (D95)	18

BMI, body mass index; D95, dose covering 95% of the PTV; ECOG, Eastern Cooperative Oncology Group; GOLD, Global Initiative for Chronic Obstructive Lung Disease; FEV, forced expiratory volume; GGN, ground-glass nodule; ITV, internal target volume; NSCLC, non-small cell lung cancer; PS, performance status; PTV, planning target volume

^†^These variables have missing values

### Treatment and disease control

**Fig. 1:**
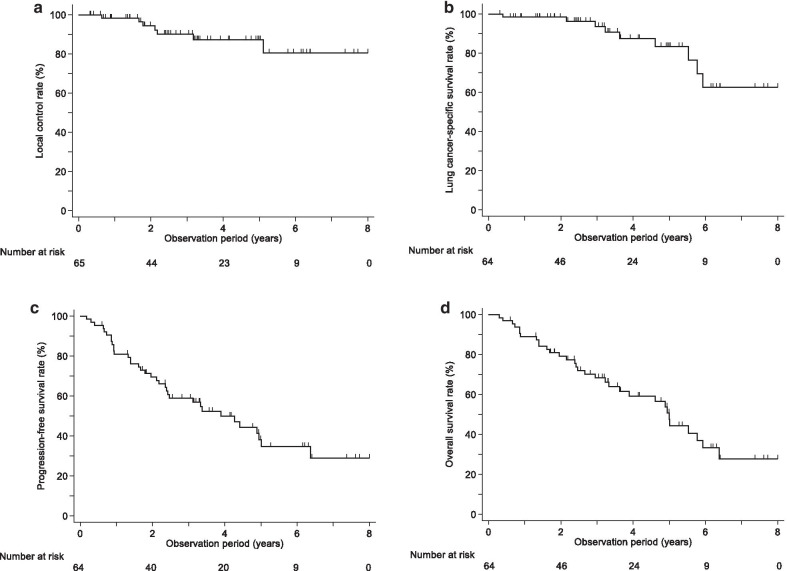
Local control and survival rates after stereotactic body radiotherapy. (**a**) Local control, (**b**) cancer-specific survival, (**c**) progression-free survival, and (**d**) overall survival

The median survival time was 60.0 months (95% confidence interval, 43.5–71.1). Forty-one patients were treated with 48 Gy to the isocenter, six were treated with 60 Gy to the isocenter, and 18 were treated with 48 Gy (D95). The median (range) PTV was 32.4 (9.6–110.6) ml. The 1-, 3-, and 5-year LC rates were 98.4%, 90.1%, and 87.4%, respectively. The 1-, 3-, and 5-year CSS, PFS, and OS rates were 98.4%, 93.7%, and 83.5%; 81.0%, 58.9%, and 38.2%; and 88.9%, 68.3%, and 47.5%, respectively (Table 2 and Fig. 1). The median time to local recurrence among the patients who developed local recurrence was 25.3 months (95% confidence interval, 7.7–38.1).

**Table 2:** Local control and survival rates after stereotactic body radiation therapy

Parameter	1-year	3-year	5-year
Local control (%)	98.4	90.1	87.4
Cancer-specific survival (%)	98.4	93.7	83.5
Progression-free survival (%)	81.0	58.9	38.2
Overall survival (%)	88.9	68.3	47.5

**Fig. 2:**
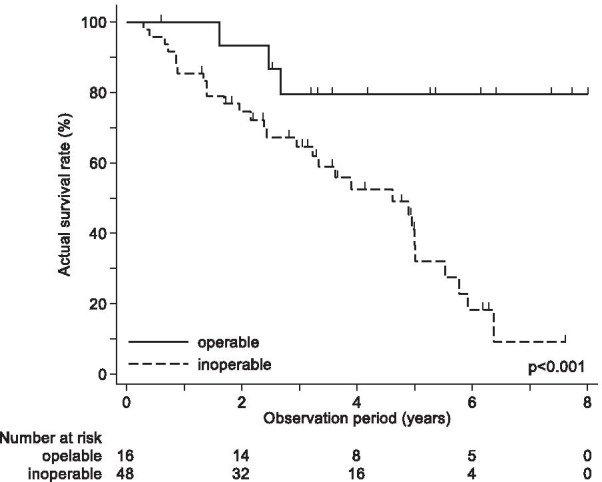
Kaplan–Meier curves of overall survival for medically operable and medically inoperable patients. Medically operable patients are represented by the solid line; medically inoperable patients are represented by the dotted line

The OS rates for operable (n = 16) and inoperable (n = 48) patients are shown in Fig. 2. The 1-, 3-, and 5-year OS rates for operable and inoperable patients were 100.0%, 79.4%, and 79.4%, and 85.4%, 64.7%, and 36.6%, respectively, with significant differences in the survival curves (log-rank test, *p* = 0.003).

Univariate analysis did not identify any predictors of LC or CSS. However, univariate analysis did identify ECOG PS, operability, tumor appearance, and tumor histology as the predictors of PFS [*p* = 0.035, 0.014, 0.021, and 0.026; hazard ratio (HR) = 1.42, 0.27, 2.65, and 2.59, respectively]. In the multivariate analysis, only operability was confirmed to be an independent predictor of a high PFS (*p* = 0.047, HR = 0.34). The results of the univariate and multivariate analyses of factors associated with OS are shown in Table 3. In the univariate analysis, sex, ECOG PS, operability, tumor appearance, and tumor histology were the predictors of OS (*p* = 0.047, 0.023, 0.008, 0.005, and 0.001; HR = 0.38, 1.53, 0.20, 5.52, and 3.14, respectively). In the multivariate analysis, operability and tumor appearance were confirmed to be the independent predictors of OS (*p* = 0.03 and 0.04; HR = 0.25 and 5.76, respectively).

**Table 3:** Univariate and multivariate analyses of overall survival

Characteristic	n	Univariate analysis			Multivariate analysis		
		HR	95% CI	*p*-value	HR	95% CI	*p*-value
Age (years)	–	1.07	0.95–1.21	0.24	–	–	–
Sex							
Male	45	1.00	–	–	1.00	–	–
Female	19	0.38	0.15–0.99	0.04^*^	1.85	0.48–7.18	0.38
BMI (kg/m^2^)	–	0.97	0.86–1.10	0.65	–	–	–
ECOG PS	–	1.53	1.06–2.21	0.02^*^	1.26	0.84–1.89	0.26
GOLD stage†							
Normal + I	42	1.00	–	–	–	–	–
II + III	17	1.50	0.72–3.11	0.28	–	–	–
FEV1.0 (L) †		0.46	0.20–1.06	0.07	–	–	–
History of lung operation							
No	50	1.00	–	–	–	–	–
Yes	14	1.49	0.68–3.24	0.32	–	–	–
Charlson Comorbidity Index	–	1.09	0.87–1.37	0.45	–	–	–
Operable							
No	48	1.00	–	–	1.00	–	–
Yes	16	0.20	0.06–0.66	0.008^**^	0.25	0.07–0.86	0.03^*^
Tumor diameter (cm)		0.99	0.64–1.52	0.96	–	–	–
ITV (mL)	–	1.00	0.97–1.03	0.96	–	–	–
PTV (mL)	–	1.00	0.98–1.01	0.83	–	–	–
T stage	–	1.16	0.88–1.53	0.29	–	–	–
Histology							
Adenocarcinoma	43	1.00	–	–	1.00	–	–
SCC + NSCLC	22	3.14	1.56–6.31	0.001^**^	1.97	0.95–4.10	0.07
Tumor appearance							
Pure GGN + part-solid GGN	20	1.00	–	–	1.00	–	–
Solid	45	5.52	1.68–18.14	0.005^**^	5.76	1.05–31.71	0.04^*^
Total dose (Gy)							
48	59	1.00	–	–	–	–	–
60	6	0.94	0.83–1.06	0.29	–	–	–
Prescribed dose							
Isocenter	47	1.00	–	–	–	–	–
D95	18	0.48	0.14–1.64	0.24	–	–	–

^*^p < 0.05; ^**^p < 0.01

BMI, body mass index; CI, confidence interval; D95, dose covering 95% of the PTV; ECOG, Eastern Cooperative Oncology Group; GGN, ground-glass nodule; GOLD, Global Initiative for Chronic Obstructive Lung Disease; FEV, forced expiratory volume; HR, hazard ratio; ITV, internal target volume; NSCLC, non-small cell lung cancer; PS, performance status; PTV, planning target volume; SCC, squamous cell carcinoma

^†^These variables have missing values

### Toxicity

All patients successfully completed SBRT. We observed grade 3 radiation pneumonitis (RP) in two patients (3.1%), while grade 5 RP was observed in one patient (1.6%). Both patients who developed grade 3 RP had been judged medically inoperable. Grade 3 RP occurred at 2.8 and 3.6 months after completing SBRT. The patient who developed grade 5 RP had been judged medically inoperable. SBRT was performed twice for bilateral lesions in the lower lobes. The patient died of respiratory failure 3.8 months after completing the second course of SBRT. The data pertaining to lung-related comorbidity, tumor location, and dosimetry of patients who developed grade ≥ 3 RP are presented in Table 4. Except for RP, other grade ≥ 3 adverse events such as rib fracture, esophageal stenosis, tracheobronchial stenosis, neuropathy, and hemorrhage were not reported.

**Table 4:** Lung-related comorbidity, tumor location, and dosimetric data of patients with radiation pneumonitis ≥ grade 3

Patient number	1	2	3
Adverse event and grade	RP grade 3	RP grade 3	RP grade 5
FEV1.0 (L)	2.2	1.2	1.3
GOLD stage	0	1	2
History of lung operation	No	No	No
Tumor location	Right lower lobe	Right lower lobe	Bilateral lower lobe
PTV (mL)	85.0	110.6	32.4/59.0
Lung V20 (%)	8.4	16.2	18.3
MLD (Gy)	6.0	8.8	10.9

FEV, forced expiratory volume; GOLD, Global Initiative for Chronic Obstructive Lung Disease; MLD, mean lung dose; PTV, planning target volume; RP, radiation pneumonitis; V20, volume receiving at least 20 Gy

## Discussion

This study confirms that SBRT is associated with high LC rates, acceptable OS rates, and low toxicity rates in elderly patients aged ≥ 80 years with pathologically proven NSCLC.

Previous studies have shown that SBRT can achieve excellent therapeutic effects with only mild adverse events [6–11, 13, 14, 20]. SBRT is less invasive than surgical resection and is performed for early-stage NSCLC in medically inoperable patients [8, 14]. While surgery is the standard treatment for patients with early-stage NSCLC who can tolerate surgical resection [4], elderly patients aged ≥ 80 years are often judged medically inoperable owing to comorbidities. Even when elderly patients are judged medically operable, surgical resection has been related to high morbidity and mortality rates [5, 20–22]. The postsurgical morbidity and mortality rates of elderly patients have been reported to be 18.0–45.0% and 0.0–15.0%, respectively [21, 23]. Compared with surgical resection, SBRT is associated with a lower morbidity rate of 1.3–5.4% in elderly patients [23–25]. Furthermore, the frequency of grade 5 SBRT-related toxicities in elderly patients has been reported to be 0.0–2.1% [10, 11, 13, 14]. In the present study, two patients (3.1%) developed grade 3 RP, and one patient developed grade 5 RP (1.6%). In the patient with grade 5 RP, non-breath-hold SBRT was performed twice for bilateral lesions in the lower lobes. Therefore, it is possible that the irradiated lung dose could have increased unexpectedly.

There are a few reports on the treatment outcomes of SBRT in elderly patients with pathologically proven NSCLC. Shu et al*.* [18] reported the outcomes of 68 patients aged ≥ 75 years with pathologically proven early-stage (T1-3N0M0) NSCLC. The 1-, 3-, and 5-year LC rates were 95.6%, 88.9%, and 85.6%, respectively. The 1-, 3-, and 5-year OS rates were 92.6%, 77.2%, and 59.1%, respectively. In this study, the 1-, 3-, and 5-year LC and OS rates were 98.4%, 90.1%, and 87.4%, and 88.9%, 68.3%, and 47.5%, respectively. Although our OS rates may appear slightly worse, we postulate that these differences may be owing to the age of the patients because our LC rates were almost identical to those of the previous report [18]. Kreinbrink et al*.* [13], Takeda et al*.* [14], and Bei et al. [15] reported the treatment outcomes of elderly patients aged ≥ 80 years with early-stage NSCLC. In the study by Kreinbrink et al*.* [13], the 1- and 2-year LC and OS rates were 100.0% and 92.3%, and 83.5% and 59.2%, respectively. In the study by Takeda et al*.* [14], the 1- and 3-year LC and OS rates were 94.4% and 82.3%, and 95.0% and 53.7%, respectively. In the study by Bei et al*.* [15], the 3-year OS rate was 65.3% [15]. Our results are comparable to those of the previous three studies. However, the previous studies included some lesions that had not been pathologically confirmed as NSCLC. Pulmonary nodules can be benign as well as malignant [16, 17]. Yang et al*.* reported the diagnostic accuracy of percutaneous transthoracic needle biopsy in the evaluation of solitary pulmonary nodules [16]. Among 311 nodules, 78 (25.1%) nodules, including organized pneumonia, tuberculosis, sclerosing hemangioma, hamartoma, and aspergillosis, were benign. SBRT for benign lesions is contraindicated and, to some extent, can affect treatment outcomes. Because local recurrence and metastasis are not observed in a benign disease, LC and PFS may be higher than those in cases with pathologically diagnosed lung cancer. Therefore, it is desirable to make a definitive pathological diagnosis before cancer treatment. Because all SBRT-treated lesions in this study had been diagnosed as NSCLC, our treatment outcomes are more reliable. To the best of our knowledge, this is the first study to include only patients aged ≥ 80 years with pathologically proven NSCLC.

There were no predictors of higher LC in this study. Previous studies showed that the biologically effective D95 was significantly correlated with the LC rate [26, 27]. However, there was no significant difference between the prescribed doses (D95 vs. isocenter) in this study. The reason for this difference may be owing to the small sample size. Kimura et al. are conducting a nationwide clinical trial on dose escalation for SBRT [28]. This study is expected to reveal whether dose escalation is also beneficial in elderly patients.

In the present study, we found operability to be an independent predictor of good OS. Takeda et al*.* [14] reported that the 3-year OS rates for operable and inoperable patients were 58.1% and 48.3%, respectively. Therefore, operability turned out to be a predictor of good OS after SBRT in elderly patients. Bei et al. [15] also demonstrated that operability was a predictor of good OS in multivariate analysis. Similarly, in our study, the 3-year OS rates for operable and inoperable patients were 79.4% and 64.7%, respectively, and operability was a significant prognostic factor in multivariate analysis (p = 0.03). Our results are comparable with those of the previous reports that have shown that medically operable patients have better prognosis. We initially considered that inoperable patients might have a shorter life expectancy than operable patients because inoperable patients had poor respiratory function, many comorbidities, or a poor general condition. Nagata et al*.* [8] reported that the 3-year OS rates for operable and inoperable patients in the Japan Clinical Oncology Group 0403 trial were 68.3% and 63.7%, respectively, and there is no difference in terms of OS between the two groups. In their study, less than one-third of the patients were aged > 81 years, and the differences in patients’ age may be the cause of these varying results. In elderly patients, operability may have a more pronounced effect on OS than in younger patients. However, further research is warranted.

Wang et al. showed that CT appearance was a significant prognostic factor for OS in patients with early-stage lung cancer who underwent surgery, with the worst prognosis for solid tumors [29]. Solid nodules were also a predictor of poor OS in our study. However, no reasonable interpretation of the OS data can be made because solid nodules were not a predictor of PFS in multivariate analysis. Since CT appearance is a significant prognostic factor for PFS in univariate analysis, this result may be owing to the small sample size of this study.

Our study has a few limitations. First, this was a retrospective, single-center study with a small sample size. Second, we did not use a quantitative model for judging operability. Instead, operability was judged based on the consensus of several thoracic surgeons or respiratory physicians. Nevertheless, the treatment outcomes showed that SBRT achieved high LC rates and acceptable OS rates, even in elderly patients aged ≥ 80 years with pathologically proven NSCLC. In addition, our results indicated that medically operable patients had better treatment outcomes. We believe that our findings may help select an appropriate treatment strategy for patients aged ≥ 80 years with NSCLC.

## Conclusions

We reviewed treatment outcomes of SBRT for elderly patients aged ≥ 80 years with pathologically proven NSCLC. Our findings suggest that SBRT is beneficial for elderly patients with early-stage NSCLC for whom it is difficult to determine the optimal treatment strategy.

## Data Availability

The datasets generated and/or analyzed during the current study are not publicly available due to the requirements of the Institutional Review Board, but are available from the corresponding author on reasonable request.

## Abbreviations

CI: Confidence interval
CSS: Cancer-specific survival
CT: Computed tomography
D95: Dose covering 95% of the PTV
ECOG: Eastern Cooperative Oncology Group
FEV: Forced expiratory volume
GGN: Ground-glass nodule
GOLD: Global Initiative for Chronic Obstructive Lung Disease
HR: Hazard ratio
ITV: Internal target volume
LC: Local control
NSCLC: Non-small cell lung cancer
OS: Overall survival
PS: Performance status
PFS: Progression-free survival
PTV: Planning target volume
RP: Radiation pneumonitis
SBRT: Stereotactic body radiation therapy
